# Sex Differences in Cortical Hemodynamic Responses During Interactive and Passive Tasks: An fNIRS Study Using the Nefroball System

**DOI:** 10.3390/s25185897

**Published:** 2025-09-20

**Authors:** Karolina Jezierska, Agnieszka Turoń-Skrzypińska, Iwona Rotter, Anna Syroka, Aleksandra Rył

**Affiliations:** 1 Department of Medical Physics, Pomeranian Medical University, ul. Ku Słońcu 13, 71-073 Szczecin, Poland; 2 Department of Medical Rehabilitation and Clinical Physiotherapy, Pomeranian Medical University in Szczecin, ul. Żołnierska 54, 71-210 Szczecin, Poland; agneszka.turon.skrzypinska@pum.edu.pl (A.T.-S.); iwona.rotter@pum.edu.pl (I.R.); anna.syroka@pum.edu.pl (A.S.); aleksandra.ryl@pum.edu.pl (A.R.)

**Keywords:** functional near-infrared spectroscopy fNIRS, sex differences, nefroball, interactive and passive tasks

## Abstract

The present study aimed to investigate sex differences in the hemodynamic response of the cerebral cortex during interactive and passive tasks using functional near-infrared spectroscopy fNIRS. Ninety-seven healthy adults (63 women, 34 men) participated in the study. Participants performed two tasks: an interactive motor game and a passive hand movement, and activation was measured in five cortical regions. Statistically significant differences in the amplitude of the hemodynamic response of oxygenated haemoglobin ΔHbO levels were observed, particularly in the parietal cortex, where men showed higher activation levels. The differences remained significant in the parietal, prefrontal, left hemisphere, and visual cortex. The differences were more pronounced in the passive task, which may indicate different processing strategies in women and men. Although no significant group differences were found in the latency time of maximum reaction t_max_, men tended to have longer times in the visual cortex. Additionally, a moderate positive correlation between ΔHbO and t_max_ was observed among men, particularly in the prefrontal cortex. These results highlight the importance of considering biological sex in neuroimaging studies and suggest directions for further analysis.

## 1. Introduction

Individual differences in human brain function, including those resulting from gender, are a significant area of research in neuroscience. They concern both responses to various types of behavioural stimuli and patterns of neuronal activation recorded using neuroimaging methods. Of particular interest are gender differences in cortical responses to cognitive, sensory, and motor tasks that require attention or movement initiation [1,2,3].

Functional near-infrared spectroscopy (fNIRS) enables non-invasive measurement of changes in oxygenated (HbO) and deoxygenated (Hb) haemoglobin concentrations in the cortex, including after exposure to specific stimuli. Its use enables the analysis of brain activity under conditions closely analogous to natural ones, making it particularly useful in studies that require mobility or simulate real-world environmental conditions [4,5,6,7]. Clinical research in neuroscience and neurology using fNIRS covers topics such as language mapping, cerebral hemodynamic changes in stroke, epilepsy diagnosis, autonomic nervous system function, migraine, mild cognitive impairment, and others [8]. In psychiatry, fNIRS has been used to diagnose and understand conditions such as anxiety disorders, affective disorders, personality disorders, schizophrenia, and the consequences of substance abuse [9]. fNIRS, of course, enables the analysis of individual differences, including gender [10]. Previous studies have shown that women and men may differ in the intensity of cortical activation, response lateralisation, and the range of brain structures involved. These differences are observed both during the resting state and during active task performance. fMRI and EEG studies indicate gender-specific differences in the involvement of frontal, parietal, and motor structures [11,12,13,14,15]. In the context of active and interactive tasks, Cazzell et al. (2012) demonstrated significant differences in prefrontal cortex activation during a risky task, and Huang et al. (2025) reported significantly higher HbO levels in men in a paired drawing task [16,17]. Gender-specific differences in brain response have also been documented in tasks using gaming environments, virtual rehabilitation, and training applications. Studies of children demonstrated significantly stronger prefrontal activation in boys than in girls during interactive tasks compared to passive tasks [18]. In turn, rehabilitation using neurofeedback and virtual environments has been shown to enhance HbO activation compared to traditional approaches, with potential modulation by individual factors, including gender [19]. A literature review confirms that game-based and touch-based control systems elicit distinct patterns of cortical activation, depending on the interaction method, which may be important in assessing the dynamics of learning and attention [20].

This study aimed to analyse the influence of gender on cortical hemodynamic activity using fNIRS in five cortical areas during both an interactive task (utilising the Nefroball system) and a passive task. Particular attention was paid to comparing the amplitude and latency of the HbO response between women and men, which allowed for the identification of potential differences in the processing of stimuli of a different motor and cognitive nature.

## 2. Materials and Methods

Participants were a homogeneous group of young adults. Therefore, demographic characteristics are presented jointly. Ninety-seven healthy volunteers (63 women and 34 men) aged 19–41 years (median = 19 years, Q1 = 19, Q3 = 20) were enrolled in the study, with no significant difference between males and females. Fifteen of the participants were left-handed. Before the experiment, each participant completed a questionnaire detailing their general well-being, any chronic diseases they suffered from (including neurological, motor, and ophthalmological conditions), and the medications they were taking. All participants provided written informed consent. The local ethics committee approved the project.

**Figure 1:**
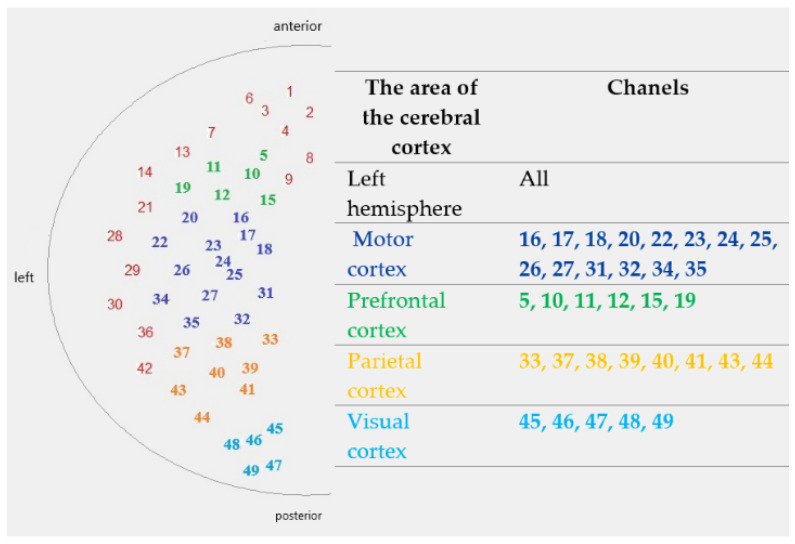
Diagram of channel arrangement on the head surface, divided into the analysed areas of the cerebral cortex and adapted from our previous publication [21].

An fNIRS system (NIRScout, NIRx Medical Technologies LLC, Glen Head, NY, USA) was used to record changes in haemoglobin absorption within the cerebral cortex. The cap was equipped with 16 near-infrared light emitters and 16 detectors. The channel system (emitter-detector pair) covered the entire left hemisphere, as shown in Figure 1. This allowed for the collection of signals from selected areas (left hemisphere of the brain): visual (occipital), parietal, motor, and prefrontal cortex. The channel system was based on the 10–20 EEG standard [20]. Measurements were conducted under minimal lighting conditions.

**Figure 2:**
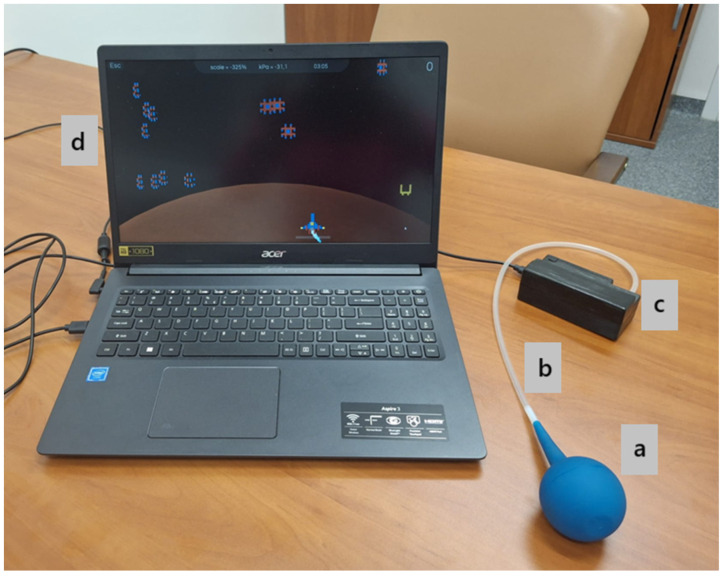
The NefroBall system. a. Pressure Controller: This spherical, pneumatic rubber device (made of SBR, NR, or silicone) features a 1 mm opening and a 2 mm PE connector. Its modular design allows for various ball sizes to accommodate different hand sizes, ensuring both airtightness and ease of disinfection. b. Connection Tube: A sealed conduit that transmits pressure changes, resulting from squeezing the ball from the controller to the measurement system. c. Measurement System: Housed in a compact unit, this system incorporates an Atmega32U4 microcontroller (Panopticum Radosław Nagay, Szczecin, Poland) and an MPX5700AP pressure sensor (Panopticum Radosław Nagay, Szczecin, Poland). It measures the force and duration of applied pressure, converting these changes into voltage and then into normalised values ranging from 0 to 1024. Voltage signals were preprocessed and normalised automatically by the NefroBall system v1.0 software according to the manufacturer’s algorithm, ensuring comparability of values across participants. Data are sampled at 25 Hz and transmitted to a computer via USB. d. Computer Application: This software module is responsible for data visualisation and storage, comprising three distinct functionalities: patient database management, standard training protocols, and gamified training, which includes a “Space Invaders” game.

The NefroBall system, a custom-built prototype developed at Pomeranian Medical University in Szczecin, Poland, was utilised in this study. This system, which is not commercially available but is protected by a Polish patent (Patent No. PL 247408; application No. P.444325, filed on 5 April 2023, granted on 23 June 2025) assigned to the Pomeranian Medical University in Szczecin, Poland, is designed to facilitate vascular access training for haemodialysis patients by enabling regular fistula exercises with remote monitoring capabilities for medical professionals. The system’s components are illustrated in Figure 2.

Previous studies using the Nefroball system have demonstrated its usefulness in assessing hemodynamic responses associated with tasks requiring active movement control and in gamified interfaces [21]. The current study builds upon these observations, supplementing them with a gender comparison and an analysis of activity in passive conditions.

Each participant wore an fNIRS cap with installed optodes. The device was calibrated before each study. Signal quality and the adhesion of the optodes to the skin surface were also checked. The participant sat in a comfortable chair, approximately 60–70 cm from the computer monitor. For the study, participants engaged in two tasks, Protocol II (Passive task) and Protocol I (Interactive task), presented in a randomised order to mitigate learning or fatigue effects. Each task lasted approximately 3 min, with a brief 1–2 min rest period in between.

- In Protocol I (Interactive task), participants performed the same ball-squeezing movement. However, in this condition, the NefroBall system registered each movement, allowing the participant to shoot down virtual objects in the “Space Invaders” game. The goal was to shoot down as many objects as possible within a set time, requiring eye-hand coordination as ball compression directly controlled the number of projectiles fired by the plane. During the interactive task, participants used the NefroBall system by pressing the ball with a predefined force threshold set in the application. Once the required pressure was reached, a virtual aeroplane on the screen fired projectiles, thereby “shooting down” the flying objects. In this way, foot-applied pressure was directly translated into an action within the game.
- In Protocol II (Passive task), participants rhythmically squeezed the pressure controller approximately every 2 s. Simultaneously, they observed a computer screen displaying the “Space Invaders” game with an aeroplane moving, but their actions did not affect the game. The objective was to maintain consistent ball compression without reacting to on-screen changes. Protocol II was a control study.

**Figure 3:**
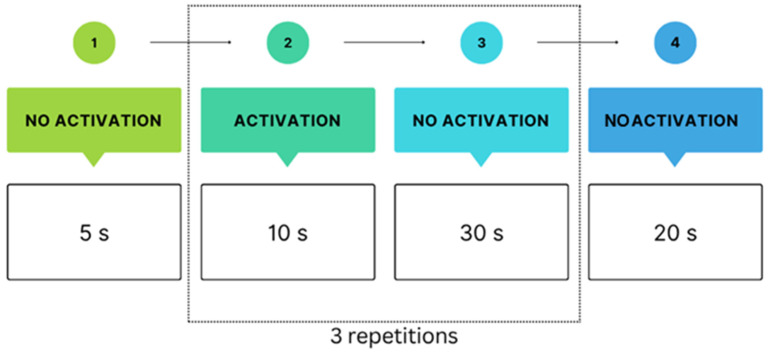
Experimental design. Each condition began with a short baseline rest, followed by three repetitions of alternating “no activation” and “activation” blocks, and ended with a final rest. “Activation” consisted of either passive observation (Protocol II) or an interactive task using the Nefroball device (Protocol I). “No activation” blocks served as rest periods to minimise carryover effects and allow the hemodynamic response to return to baseline between tasks.

Both Protocols, I and II, followed the same study design as depicted in Figure 3 To minimise motion artefacts, participants were instructed to limit movement, and the fNIRS cap was additionally stabilised with tape.

Data were analysed using NIRSLab 15.0 software. The signal was filtered (bandpass 0.01–0.2 Hz). Channels with noisy signals were manually excluded. The dynamics of HbO changes were analysed in the conducted studies. For each study, the following was determined:

- The amplitude of the hemodynamic response, ΔHbO, which is the difference between the maximum (after the stimulus) and the minimum (before the stimulus) of the signal for HbO
- Latency time (t_max_), which is the time from the onset of the stimulus (beginning of task execution) to the maximum HbO signal.

The Shapiro–Wilk test was used to assess the distribution of the variables. Due to the lack of normal distribution of the obtained data (Shapiro–Wilk), comparisons of medians were made using the Mann–Whitney test. The relationship between ΔHbO and t_max_ was assessed using the Spearman test. A level of *p* < 0.05 was considered significant (Statistica 13, Dell Inc., Round Rock, TX, USA).

## 3. Results

**Figure 4:**
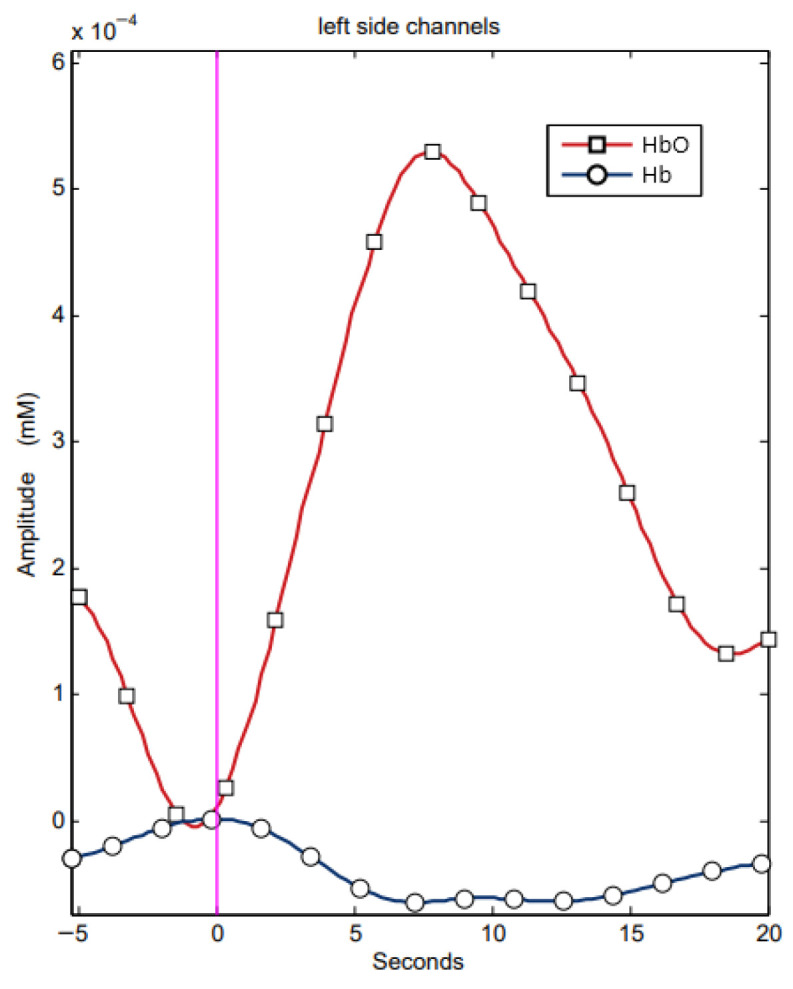
Examples of fNIRS signal recordings from channels located in the left hemisphere. The x-axis represents time (in seconds), while the y-axis shows changes in oxyhemoglobin and deoxyhemoglobin concentration expressed in mmol/L.

None of the participants reported any symptoms that could affect motor or cognitive function. All participants were neurologically healthy, with no history of epilepsy, brain injury, or significant visual impairment. Figure 4 shows an example recording of the fNIRS signal.

**Figure 5:**
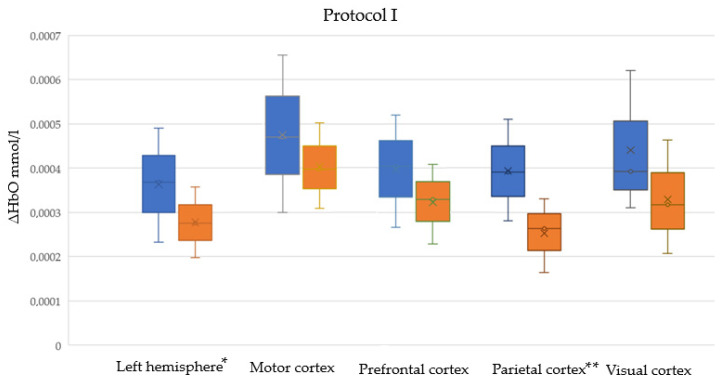
Sex-related differences in cortical hemodynamic responses (ΔHbO) across five cortical regions during Protocol I (interactive task). Boxplots show distributions for male (blue) and female (orange) participants. Crosses denote the mean values. Statistically significant differences are indicated with asterisks placed next to the labels of the corresponding cortical regions on the x-axis (* *p* < 0.05; ** *p* < 0.01).

**Figure 6:**
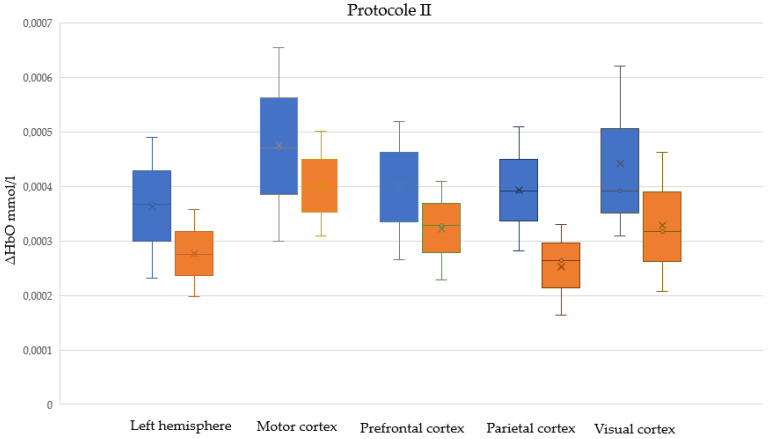
Sex-related differences in cortical hemodynamic responses (ΔHbO) across five cortical regions during Protocol II (passive task). Boxplots show distributions for male (blue) and female (orange) participants. Crosses denote the mean values. All observed differences between groups were statistically significant.

**Figure 7:**
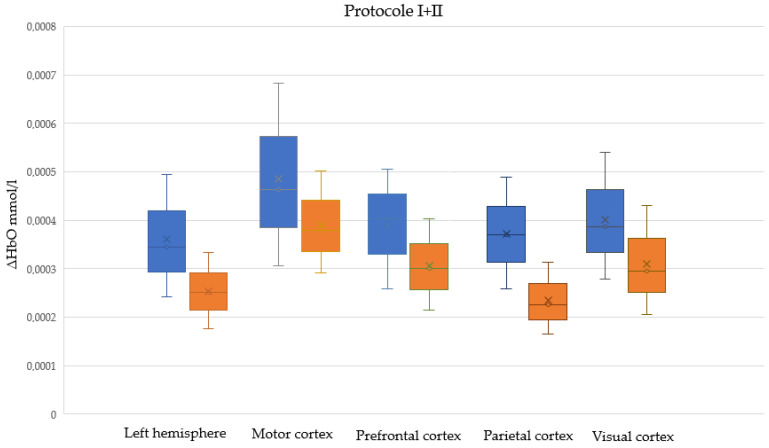
Sex-related differences in cortical hemodynamic responses (ΔHbO) across five cortical regions during combined Protocol II (passive task)+ Protocol I (interactive task). Boxplots show distributions for male (blue) and female (orange) participants. Crosses denote the mean values. All observed differences between groups were statistically significant.

Tables S1 and S2 present descriptive statistics for ΔHbO and t_max_ among male and female participants, respectively, in five cortical regions, in Protocols I, II, and I + II (combined). Normal distributions were assessed using the Shapiro–Wilk test; the table presents the W statistic and the corresponding *p*-values. Figure 5, Figure 6 and Figure 7 illustrate male and female differences in ΔHbO across cortical regions for Protocols I, II, and I + II, respectively. These plots are provided only for ΔHbO, as no statistically significant sex-related differences were observed for t_max_ in any of the regions.

**Figure 8:**
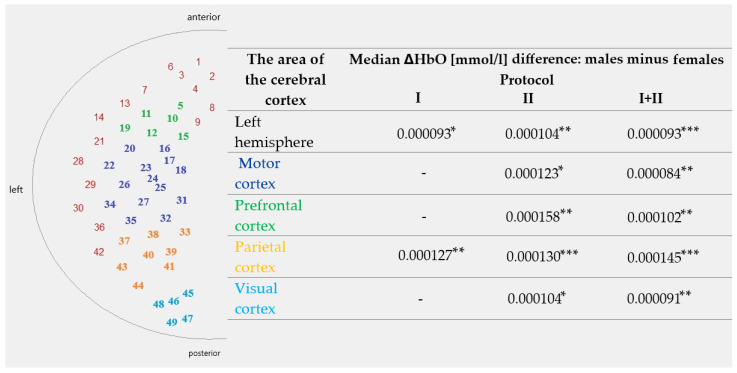
Sex-related differences in cortical activation (ΔHbO) across regions of the left hemisphere. The schematic diagram (**left** panel) shows optode positions grouped into regions of interest: prefrontal, motor, parietal, visual, and the hemisphere as a whole. The table (**right** panel) presents median differences (males minus females) in ΔHbO [mmol/l] for each protocol. Asterisks indicate statistically significant differences (* *p* < 0.05; ** *p* < 0.01; *** *p* < 0.001).

Table 1 and Table 2 present the results of the comparison of ΔHbO and t_max_, respectively, between men and women in different areas of the cerebral cortex for two Protocols (I and II) and their combination (I + II). Data are reported as the median difference (men minus women), with *p*-value and Mann–Whitney U-test statistic. Figure 8 summarises the sex-related differences in ΔHbO across cortical regions. The schematic diagram on the left shows the spatial arrangement of optodes and areas of interest, while the table presents median differences (males minus females) for Protocols I, II, and I + II. Asterisks denote the level of statistical significance (* *p* < 0.05, ** *p* < 0.01, *** *p* < 0.001). These results are presented only for ΔHbO, as no statistically significant sex-related differences were found for t_max_.

Table 3 and Table 4 present the correlation analyses (R coefficient and its corresponding *p*-value) between ΔHbO and t_max_ for women and men, respectively, in different brain regions for two Protocols (I and II) and their combination (I + II).

## 4. Discussion

This study aimed to identify sex-related differences in cortical hemodynamic responses during passive and interactive tasks using the Nefroball system. Recordings were performed using fNIRS, analysing changes in oxygenated haemoglobin concentration (ΔHbO) and time to peak response (t_max_) in five regions of the cerebral cortex.

The obtained results revealed statistically significant differences between women and men, particularly in the parietal cortex. Differences in ΔHbO (men > women) remained important in the left hemisphere (all Protocols), motor cortex (Protocol II and combined Protocols I + II), prefrontal cortex (Protocol II and combined Protocols I + II), parietal cortex (all Protocols), and visual cortex (Protocol II and combined Protocols I + II). Among the examined regions, the parietal cortex showed the most consistent and robust sex-related differences, with men exhibiting higher ΔHbO across all protocols. This pattern suggests that parietal activation may represent the strongest marker of sex-related variability in cortical hemodynamic responses within the current experimental paradigm. Notably, the parietal cortex not only demonstrated significant differences across all protocols but also showed the most considerable median differences between men and women and the lowest *p*-values among all analysed regions. At the same time, additional condition-dependent effects were observed in the prefrontal and motor cortices, as well as in the visual cortex and overall left hemisphere activity. These findings, while less consistent than in the parietal cortex, further highlight the distributed nature of sex-related variability in cortical activation. This supports the view that parietal activation constitutes the most prominent and reliable marker of sex-related differences in the present study. These results are consistent with previous studies, which have shown greater activation of the prefrontal and parietal cortices in men during tasks requiring attention, visuomotor coordination, or interaction [12,13,14]. Interestingly, data analysis reveals a clear trend in which gender differences were more pronounced in Protocol II (the passive task) than in Protocol I, which is interactive. In Protocol I, significant differences were observed only in the parietal cortex. In contrast, in Protocol II, differences also appeared in the prefrontal cortex, the left hemisphere, and the visual cortex. This may suggest that passive tasks more strongly reveal differences in the way stimuli are processed between women and men, perhaps in the context of internal engagement, information processing strategies, or responses to a lack of control over the stimulus.

Particularly significant differences were observed in the parietal cortex, which is responsible for integrating visuospatial information and planning movement. Similar observations were made by Ingalhalikar et al. [12] and Frederikse et al. [13], who indicated marked differences in structural connectivity between the sexes, particularly in parietal regions. Cazzell et al. [16] observed differences in prefrontal cortex activation during risky or interactive tasks, findings similar to our own.

Statistically significant differences in t_max_ were observed in the visual cortex, where men showed longer peak signal times in Protocol I and in the combined analysis (I + II). These results suggest that men may require more time to reach maximum cortical activation in response to visual stimuli, which may reflect differences in the temporal dynamics of stimulus processing or in functional blood supply between sexes. Furthermore, significant correlations between ΔHbO and t_max_ were found in the motor cortex among women (Protocol II) and in the prefrontal cortex among men (Protocol II and I + II). These associations may indicate sex-specific differences in neurovascular coupling, consistent with observations described by Scholkmann et al. [4]. Notably, the prefrontal cortex in men showed the strongest relationship, suggesting that higher hemodynamic responses were linked to longer peak latencies, which could reflect different strategies of cortical engagement.

From a translational perspective, these results highlight the necessity of considering sex differences in designing rehabilitation or neurofeedback-based interventions. Hemodynamic activation patterns that differ between women and men suggest that interactive, game-based tasks may need to be personalised to optimise cortical engagement. For instance, enhanced IPL and prefrontal recruitment in men could be leveraged in visuomotor training, whereas women might benefit from strategies engaging broader cortical integration. Such sex-specific approaches may ultimately improve the efficacy of personalised rehabilitation protocols using fNIRS and interactive systems such as the NefroBall. In addition to structural and functional differences, sex hormones may also contribute to the observed variability, given their established role in modulating cerebral blood flow and neurovascular coupling. Moreover, these findings underline the methodological importance of accounting for sex in fNIRS analyses, as ignoring sex-related variability may obscure meaningful effects in group-level comparisons. Future studies should therefore consider both hemispheres, larger and balanced cohorts, and the potential role of hormonal status (e.g., menstrual cycle in women), to further clarify the biological underpinnings of sex-specific cortical responses.

Several limitations that may influence the interpretation of the results should be emphasised. First, recordings were limited to the left hemisphere, which limits the complete picture of activation, especially in the context of the lateralisation of cognitive and motor functions. It is well established that gender differences in lateralisation are significant; for example, a study by Shaywitz et al. [11] demonstrated apparent differences in the linguistic organisation of the cerebral hemispheres between women and men. Further studies are planned to examine both hemispheres in this regard. Second, the unequal sample sizes (63 women vs. 34 men) may affect the statistical power of the tests and the equality of variances. Although the analysis employed nonparametric tests, future studies should strive for a more balanced sample size. Third, like any fNIRS study, this one is also limited to analysing signals originating primarily from cortical layers, without the ability to precisely assess deep brain structures. An additional limitation of the present study is that the potential effects of age within the male and female groups were not investigated. The sample consisted mainly of young adults within a relatively narrow age range, which likely reduces but does not exclude the influence of developmental factors. Future studies including broader and more balanced age groups, as well as measures of pubertal stage, are needed to address possible age-related effects on cortical activation. Another limitation of the present study is that parameters related to pubertal stage (e.g., Tanner stage, bone age, or biochemical markers) were not assessed. Although the participants were young adults (median age 19 years, range 19–41), it cannot be entirely excluded that developmental factors related to the end of puberty may have influenced cortical activation. This aspect should be addressed in future studies involving broader and age-balanced samples with a dedicated assessment of pubertal stage.

Despite these limitations, the study provides significant evidence that biological sex influences patterns of brain activation during tasks of various motor and cognitive nature. In the context of fNIRS, this is particularly important, as many analyses implicitly assume homogeneity of hemodynamic responses across the population. However, as Cahill [2,15] notes, gender differences are not noise but biologically determined variability that should be accounted for in neuroimaging analyses.

These sex-related differences may also have important clinical implications. For example, in neurological disorders such as stroke, traumatic brain injury, or neurodegenerative diseases, rehabilitation outcomes could be influenced by sex-specific patterns of cortical engagement. Considering such variability may therefore be crucial in tailoring neurofeedback protocols or game-based interventions for clinical populations.

## 5. Conclusions

The study found significant differences in hemodynamic activation of the cerebral cortex between women and men during interactive and passive tasks. Men demonstrated higher ΔHbO values in several areas, particularly the parietal cortex, which may indicate differences in attentional engagement, execution strategies, or neurovascular coupling. Differences in the time to peak response were less consistent but reached significance in the visual cortex, suggesting sex-related variability also in the temporal dynamics of cortical activation.

The obtained results emphasise the need to consider gender as a biological variable in fNIRS research, both in scientific and clinical contexts. This is particularly important in designing personalised rehabilitation programs or neurofeedback training based on interactive activities. Future research recommends extending measurements to both hemispheres, employing more balanced study samples, and analysing interactions between gender and task difficulty or stimulus type.

## Figures and Tables

**Table 1:** Median differences in ΔHbO between male and female participants (males minus females) across cortical regions of the left hemisphere under three Protocol conditions (I, II, and I + II combined). The table includes *p*-values and U statistics from the Mann–Whitney U test. Statistical significance was defined as *p*-value < 0.05.

The Area of the Cerebral Cortex	Protocol	Median ΔHbO [mmol/L] Difference: Males Minus Females	*p*-Value	U Test Statistic
Left hemisphere	I	0.000093	0.022	741
	II	0.000104	0.001	599
	I + II	0.000093	<0.001	2695
Motor cortex	I	0.000072	0.165	831
	II	0.000123	0.014	694
	I + II	0.000084	0.009	3098
Prefrontal cortex	I	0.000075	0.060	755
	II	0.000158	0.004	631
	I + II	0.000102	0.001	2757
Parietal cortex	I	0.000127	0.001	473
	II	0.000130	<0.001	456
	I + II	0.000145	<0.001	1870
Visual cortex	I	0.000076	0.065	576
	II	0.000104	0.014	514
	I + II	0.000091	0.002	2186

**Table 2:** Median differences in t_max_ between male and female participants (males minus females) across cortical regions of the left hemisphere under three Protocol conditions (I, II, and I + II combined). The table includes *p*-values and U statistics from the Mann–Whitney U test. Statistical significance was defined as *p*-value < 0.05.

The Area of the Cerebral Cortex	Protocol	Median t_max_ [s] Difference: Males Minus Females	*p*-Value	U Test Statistic
Left hemisphere	I	0.125	0.892	1019
	II	0.375	0.516	953
	I + II	0.625	0.589	3951
Motor cortex	I	0.125	0.732	964
	II	0.250	0.579	937
	I + II	0.250	0.768	3926
Prefrontal cortex	I	0.250	0.621	928
	II	0.750	0.492	904
	I + II	0.500	0.429	3682
Parietal cortex	I	−0.125	0.377	742
	II	2.125	0.391	759
	I + II	0.500	0.917	3357
Visual cortex	I	−1.000	0.029	541
	II	−0.875	0.244	646
	I + II	−3.250	0.014	2355

**Table 3:** Correlation coefficients (R) and *p*-values for the relationship between changes in ΔHbO and t_max_ in female participants, across cortical regions and Protocol conditions (I, II, I + II combined). Only *p*-values < 0.05 were considered statistically significant.

Female			
The Area of the Cerebral Cortex	Correlation Analysis Between ΔHbO and t_max_ for Protocols:	R	*p*-Value
Left hemisphere	I	0.08	0.555
	II	0.21	0.103
	I + II	0.10	0.257
Motor cortex	I	0.14	0.271
	II	0.25	0.049
	I + II	0.17	0.052
Prefrontal cortex	I	0.13	0.321
	II	0.23	0.086
	I + II	0.12	0.196
Parietal cortex	I	0.03	0.857
	II	0.13	0.342
	I + II	0.04	0.700
Visual cortex	I	0.24	0.087
	II	−0.14	0.340
	I + II	0.04	0.695

**Table 4:** Correlation coefficients (R) and *p*-values for the relationship between changes in ΔH-bO and t_max_ in male participants, across cortical regions and Protocol conditions (I, II, I + II combined). Only *p*-values < 0.05 were considered statistically significant.

Male			
The Area of the Cerebral Cortex	Correlation Analysis Between ΔHbO and t_max_ for Protocols:	R	*p*-Value
Left hemisphere	I	0.26	0.139
	II	−0.04	0.830
	I + II	0.15	0.221
Motor cortex	I	0.18	0.317
	II	0.21	0.254
	I + II	0.23	0.070
Prefrontal cortex	I	0.26	0.144
	II	0.38	0.031
	I + II	0.27	0.030
Parietal cortex	I	0.25	0.190
	II	0.01	0.970
	I + II	0.07	0.615
Visual cortex	I	0.29	0.121
	II	0.10	0.614
	I + II	0.15	0.237

## Data Availability

Data supporting reported results can be found at https://doi.org/10.7910/DVN/658FCJ, (accessed on 13 July 2025).

## Supplementary Materials

The following supporting information can be downloaded at https://www.mdpi.com/article/10.3390/s25185897/s1: Table S1: Descriptive statistics for ΔHbO and t_max_ in male participants across left-hemisphere cortical ROIs under Protocol I, II and I + II. Values are median (IQR); Shapiro–Wilk W and *p* are reported; *p* < 0.05 indicates deviation from normality; Table S2: Descriptive statistics for ΔHbO and t_max_ in female participants across left-hemisphere cortical ROIs under Protocol I, II and I + II. Values are median (IQR); Shapiro–Wilk W and *p* are reported; *p* < 0.05 indicates deviation from normality.

## Abbreviations

The following abbreviations are used in this manuscript:

fNIRS	Functional near-infrared spectroscopy
t_max_	The time from the beginning of the task to the HbO signal reaching its maximum value
HbO	Oxygenated reduced haemoglobin
Hb	Reduced haemoglobin
ΔHbO	The average amplitude of the increase in oxygenated oxyhaemoglobin
EEG	Electroencephalopathy
