# Supplementary material for: Sex Differences in Cortical Hemodynamic Responses During Interactive and Passive Tasks: An fNIRS Study Using the Nefroball System
Source: Sensors (Basel). 2025 Sep 20;25(18):5897. doi: 10.3390/s25185897 (PMC12473777; doi:10.3390/s25185897)
Supplement: Supplementary file 1 [file sensors-25-05897-s001.zip › sensors-3829188-supplementary.pdf]

Table S1. Descriptive statistics for changes in ΔHbO and tmax in male participants across different cortical areas of the left hemisphere, recorded under three Protocol conditions (I, II, and I+II combined). Medians and interquartile ranges (25th and 75th percentiles) are presented, along with Shapiro-Wilk test results (W and corresponding p-values) used to assess the normality of data distribution. p-values < 0.05 indicate significant deviations from normality.

The area of the cerebral cortex	Male	ΔHbO [mmol/l]		t_{max} [s]		ΔHbO [mmol/l]	t_{max} [s]
		Protocol		Protocol		Protocol	
		I	II	I	II	I+II	I+II
Left hemisphere	N	34	34	34	34	68	68
	Median	0,000368	0,000335	5,4	6,6	0,000345	6,1
	25th percentile	0,000232	0,000246	4,5	5,3	0,000242	4,8
	75th percentile	0,000490	0,000500	6,5	10,0	0,000495	8,5
	W	0,93	0,96	0,87	0,96	0,95	0,94
	p-value	0,040	0,213	<0,001	0,319	0,014	0,003
Motor cortex	N	32	32	32	32	64	64
	Median	0,000470	0,000460	5,6	6,8	0,000464	6,1
	25th percentile	0,000300	0,000324	5	5	0,000307	5
	75th percentile	0,000655	0,000690	7,1	8,6	0,000684	7,9
	W	0,93	0,94	0,81	0,96	0,94	0,91
	p-value	0,031	0,086	<0,001	0,259	0,003	<0,001
Prefrontal cortex	N	33	33	33	33	66	66
	Median	0,000404	0,000398	5,5	6,8	0,000403	6,1
	25th percentile	0,000266	0,000249	4,5	5,3	0,000258	5,0
	75th percentile	0,000520	0,000503	6,5	8,3	0,000505	7,8
	W	0,90	0,97	0,86	0,85	0,95	0,84
	p-value	0,005	0,549	<0,001	<0,001	0,006	<0,001
Parietal cortex	N	30	30	30	30	60	60
	Median	0,000391	0,000335	4,8	7,4	0,00037	5,5
	25th percentile	0,000281	0,000247	3,5	4,5	0,000259	3,9
	75th percentile	0,000510	0,000490	6,0	10,5	0,000489	8,9
	W	0,95	0,95	0,92	0,92	0,97	0,88
	p-value	0,176	0,225	0,023	0,024	0,088	<0,001
Visual cortex	N	30	30	30	30	60	60
	Median	0,000392	0,000377	5,0	8,6	0,000387	5,5
	25th percentile	0,000310	0,000218	3,5	4,0	0,000279	3,8
	75th percentile	0,000621	0,000490	7,0	10,8	0,000540	10,0
	W	0,95	0,94	0,93	0,93	0,96	0,93
	p-value	0,209	0,102	0,049	0,047	0,058	0,002

Table S2. Descriptive statistics for changes in ΔHbO and tmax in female participants across dif-ferent cortical areas of the left hemisphere, recorded under

three Protocol conditions (I, II, and I+II combined). Medians and interquartile ranges (25th and 75th percentiles) are presented, along with Shapiro-Wilk test results (W and corresponding p-values) used to assess the normality of data distribution. p-values < 0.05 indicate significant deviations from normality.

**The area of the cerebral cortex**	Female	ΔHbO [mmol/l]		t_{max} [s]		ΔHbO [mmol/l]	t_{max} [s]
		Protocol		Protocol		Protocol	
		I	II	I	II	I+II	I+II
Left hemisphere	N	61	61	61	61	122	122
	Median	0,000276	0,000231	5,3	6,3	0,000252	5,5
	25th percentile	0,000198	0,000169	4,3	4,8	0,000177	4,5
	75th percentile	0,000358	0,000306	8,8	9,5	0,000333	9,0
	W	0,95	0,96	0,90	0,91	0,95	0,90
	p-value	0,011	0,029	<0,001	<0,001	<0,001	<0,001
Motor cortex	N	63	63	63	63	126	126
	Median	0,000398	0,000337	5,5	6,5	0,000380	5,9
	25th percentile	0,000309	0,000278	4,8	5,3	0,000292	5
	75th percentile	0,000502	0,000503	8,8	10,8	0,000502	9,8
	W	0,97	0,96	0,88	0,89	0,97	0,88
	p-value	0,117	0,047	<0,001	<0,001	0,017	<0,001
Prefrontal cortex	N	60	60	60	60	120	120
	Median	0,000329	0,000240	5,3	6	0,000301	5,6
	25th percentile	0,000229	0,000201	4,4	4,9	0,000214	4,5
	75th percentile	0,000409	0,000340	6,5	10	0,000404	8,8
	W	0,97	0,93	0,87	0,90	0,96	0,89
	p-value	0,211	0,002	<0,001	<0,001	<0,001	<0,001
Parietal cortex	N	56	56	57	57	113	113
	Median	0,000264	0,000205	4,9	5,3	0,000225	5,0
	25th percentile	0,000164	0,000165	3,6	4,0	0,000165	4,0
	75th percentile	0,000330	0,000280	9,0	10,5	0,000313	10,0
	W	0,92	0,88	0,94	0,85	0,93	0,86
	p-value	0,001	<0,001	0,005	<0,001	<0,001	<0,001
Visual cortex	N	51	51	51	51	102	102
	Median	0,000317	0,000273	6,0	9,5	0,000296	8,8
	25th percentile	0,000207	0,000180	4,3	4,8	0,000206	4,5
	75th percentile	0,000463	0,000350	10,8	11,3	0,000430	10,8
	W	0,91	0,93	0,90	0,91	0,92	0,90
	p-value	0,001	0,006	<0,001	<0,001	<0,001	<0,001
